# Near-complete clinical improvement of an extensive congenital acantholytic epidermal nevus treated with topical fluocinolone acetonide/hydroquinone/tretinoin (Tri-luma): A case report

**DOI:** 10.1016/j.jdcr.2026.03.041

**Published:** 2026-03-27

**Authors:** Stefania Rivera Ocampo

**Affiliations:** Dermatology Resident, Colombia

**Keywords:** acantholytic epidermal nevus, case report, epidermal nevus, genodermatoses, Tri-Luma, topical therapy

Acantholytic epidermal nevus is a rare histopathologic variant of epidermal nevus characterized by focal acantholysis within the epidermis. These lesions are typically congenital, may follow Blaschko lines, and often present as hyperkeratotic, hyperpigmented plaques.^1^^,^^2^ Therapeutic options are limited and frequently invasive, including surgical excision and laser-based modalities, with variable outcomes and a significant risk of scarring.^3^^,^^4^

We report the case of an 18-year-old male with an extensive congenital acantholytic epidermal nevus involving the right axillary region with extension to the posterior trunk and anterior torso. The lesion was noted within the first days of life and progressively enlarged over time. Clinically, the nevus was characterized by verrucous, scaly, and hyperkeratotic plaques with intermittent fissuring, pruritus, and burning sensations, particularly in the axillary region (Fig 1). Histopathologic examination previously confirmed the diagnosis of acantholytic epidermal nevus.

**Fig 1:**
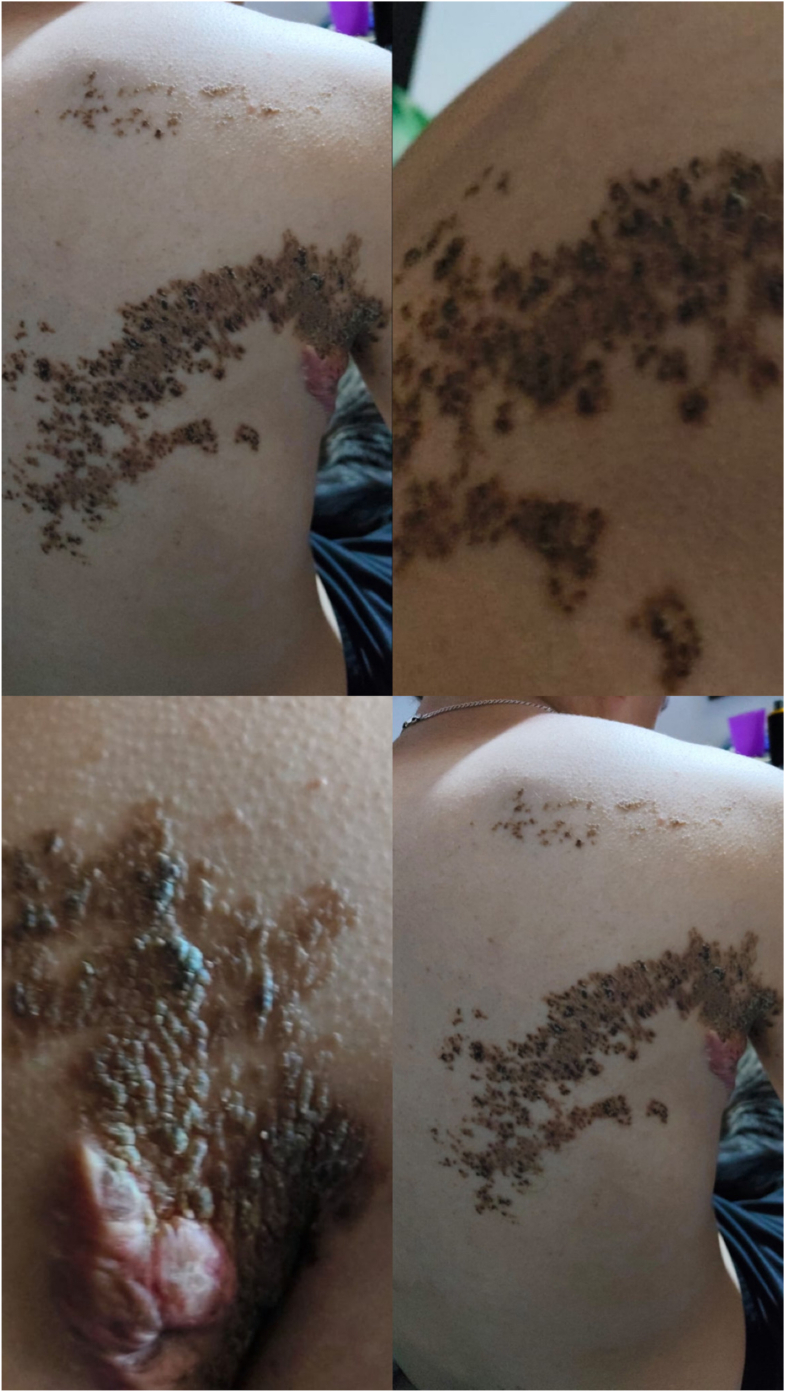
Clinical appearance of an extensive congenital acantholytic epidermal nevus involving the right axillary region and adjacent trunk before treatment.

The patient was treated with topical fluocinolone acetonide/hydroquinone/tretinoin (Tri-luma) cream applied once daily for 4 consecutive months, followed by maintenance therapy twice weekly in combination with barrier repair therapy using a ceramide-based moisturizer. Marked clinical improvement was observed, with an estimated 90% reduction in lesion thickness, scaling, and pigmentation (Fig 2). Mild transient erythema and irritation were noted as adverse effects, which resolved without treatment discontinuation.

**Fig 2:**
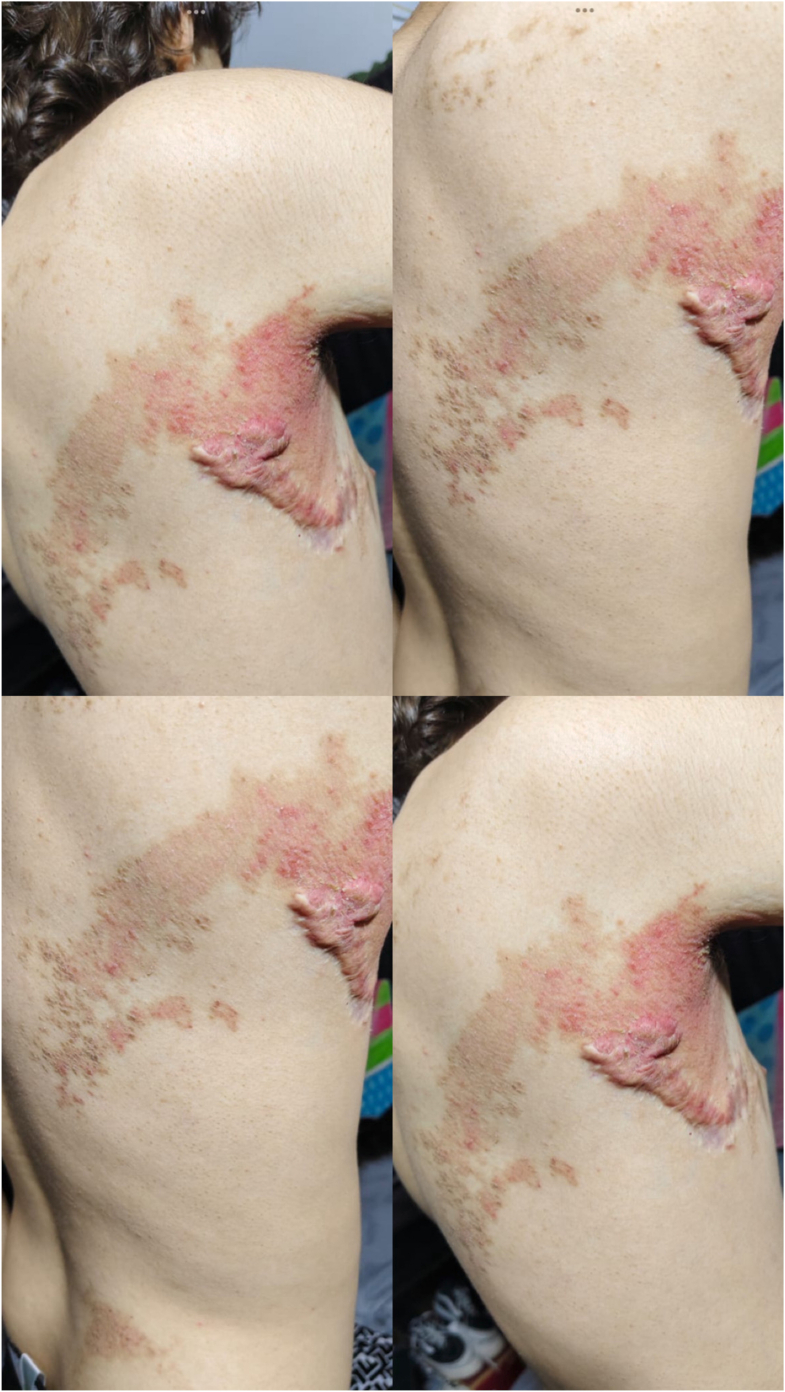
Near-complete clinical improvement after treatment with topical fluocinolone acetonide/hydroquinone/tretinoin (Tri-luma), with residual findings limited to a postsurgical axillary keloid.

In addition, intralesional triamcinolone acetonide (Kenacort-A, 10 mg/mL) was administered at 3-month intervals for a total of 3 sessions to treat a postsurgical axillary keloid resulting from a prior partial excision. At 1-year follow-up, the treated areas remained stable for 3 months, with residual findings limited to the keloid, which continues under management.

This case highlights a near-complete clinical response of an extensive congenital acantholytic epidermal nevus to a noninvasive topical regimen. To our knowledge, such a marked response to topical fluocinolone acetonide/hydroquinone/tretinoin has not been previously reported. This observation suggests that selected cases of epidermal nevus may benefit from conservative topical therapy, potentially avoiding invasive procedures and associated morbidity.

## Conflicts of interest

None disclosed.

## References

[1] Happle R. (2010). Epidermal nevi and related disorders. J Am Acad Dermatol.

[2] Vaughn O.A., Fiala E.M., Meehan S.A. (2015). Acantholytic dyskeratotic epidermal nevus: a rare variant of epidermal nevus. JAMA Dermatol.

[3] Patrizi A., Neri I., Fiorentini C. (2016). Epidermal nevi: a clinical review of treatment options. Acta Derm Venereol.

[4] Sardana K., Garg V.K. (2013). Epidermal nevi: an overview of clinical features and management. Indian J Dermatol Venereol Leprol.

